# Demonstration of Steep Switching Behavior Based on Band Modulation in WSe_2_ Feedback Field-Effect Transistor

**DOI:** 10.3390/nano14201667

**Published:** 2024-10-17

**Authors:** Seung-Mo Kim, Jae Hyeon Jun, Junho Lee, Muhammad Taqi, Hoseong Shin, Sungwon Lee, Haewon Lee, Won Jong Yoo, Byoung Hun Lee

**Affiliations:** 1 Center for Semiconductor Technology Convergence, Department of Electrical Engineering, Pohang University of Science and Technology, Cheongam-ro 77, Nam-gu, Pohang 37673, Gyeongbuk, Republic of Korea; kimsm2110@postech.ac.kr (S.-M.K.); jhjun@postech.ac.kr (J.H.J.); jhlee21@postech.ac.kr (J.L.); haewon2612@postech.ac.kr (H.L.); 2 Department of Nano Science and Engineering, Sungkyunkwan University, 2066, Seobu-ro, Jangan-gu, Suwon 16419, Gyeonggi-do, Republic of Korea; mtaqi_baiq@naver.com (M.T.); shs98753@skku.edu (H.S.); physw35@gmail.com (S.L.); yoowj@skku.edu (W.J.Y.)

**Keywords:** feedback FET, 2D materials, WSe_2_, band modulation, p−n homojunction, oxygen plasma treatment

## Abstract

Feedback field-effect transistors (FBFETs) have been studied to obtain near-zero subthreshold swings at 300 K with a high on/off current ratio ~10^10^. However, their structural complexity, such as an epitaxy process after an etch process for a Si channel with a thickness of several nanometers, has limited broader research. We demonstrated a FBFET using in-plane WSe_2_ p−n homojunction. The WSe_2_ FBFET exhibited a minimum subthreshold swing of 153 mV/dec with 30 nm gate dielectric. Our modeling-based projection indicates that the swing of this device can be reduced to 14 mV/dec with 1 nm EOT. Also, the gain of the inverter using the WSe_2_ FBFET can be improved by up to 1.53 times compared to a silicon CMOS inverter, and power consumption can be reduced by up to 11.9%.

## 1. Introduction

Rapid advances in integrated circuit (IC) technology have inevitably been accompanied by various challenges, such as a worse leakage current and high standby power consumption [1,2,3]. Various countermeasures have been introduced to alleviate these problems, but they have been limited by the physical mechanisms inherent in MOSFETs, particularly by the theoretical limitation (e.g., the thermionic emission process) of subthreshold swing with a minimum of ~60 mV/dec at room temperature [2].

Steep switching devices with novel operating mechanisms have been explored to overcome the theoretical limitations of MOSFETs’ operation [4,5,6,7,8,9,10,11,12,13,14,15,16,17]. In particular, Si channel feedback field-effect transistors (FBFETs) have attracted attention for their near-zero subthreshold swing (~0 mV/dec at 300 K) and their high on/off current ratio (~10^10^) [18,19,20,21,22,23,24,25,26,27,28,29,30,31,32]. The device structure of the FBFET includes a channel region divided into gated and ungated regions on the top of the intrinsic silicon on the oxide (SOI) body and the back gate on the bottom of the buried oxide (BOX) [33,34,35,36]. Although swing values lower than 60 mV/dec have been demonstrated for optimized FBFETs, their structural complexity, such as the epitaxy process after the etching process for channels a few nanometers thick, has significantly limited broader research.

Another approach to overcome this structural complexity is using 2D material-based homojunctions. Especially, WSe_2_ has been attracting attention as a 2D material capable of implementing a p–n homojunction [37,38,39,40,41].

In this paper, we report on a FBFET using an in-plane WSe_2_ p–n homojunction. Our device exhibited a minimum SS value of 153 mV/dec at room temperature with an on/off ratio of ~10^3^. Even though this value appears to be higher than the lower limit of the swing of a silicon MOSFET (~60 mV/dec), the abrupt transition from the off to the on state exhibited by this device demonstrates that positive feedback by potential barriers and potential wells is feasible in 2D material-based channels, as well as in Si channels, and the absolute value of swing can be scaled down by reducing the thickness of the gate dielectric. We show that the swing of the WSe_2_ FBFET can be scaled down to 14 mV/dec by reducing the equivalent oxide thickness (EOT) of the gate dielectric down to 1 nm. The theoretical projection of the performance of the inverter using the scaled WSe_2_ FBFET demonstrated up to 1.53 times higher gain and 11.9% lower power consumption compared to those of a silicon CMOS inverter. These results indicate that the WSe_2_ FBFET can be useful in targeting low-power applications.

## 2. Experiments

**Figure 1:**
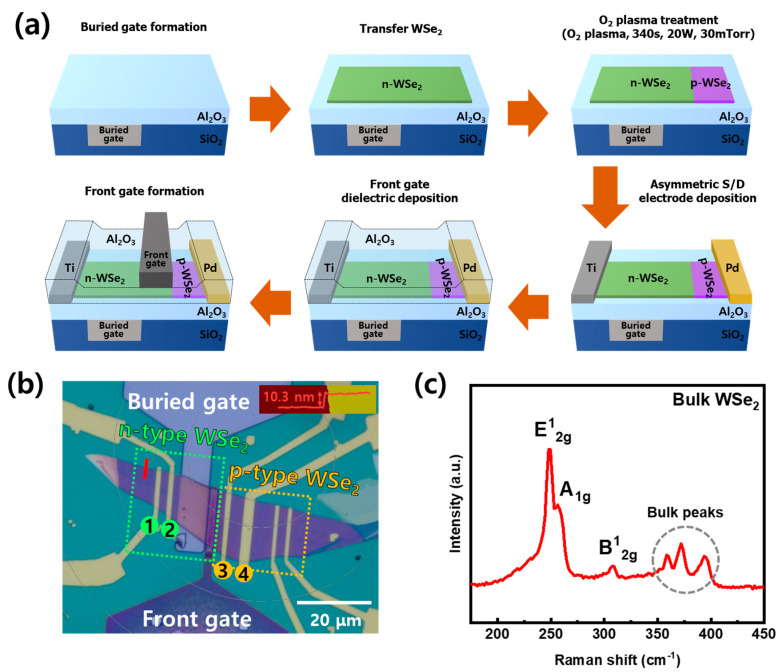
(**a**) Fabrication process of WSe_2_ FBFET. (**b**) Optical image of top view of WSe_2_ FBFET. This device includes not only FBFET but also n-/p-type WSe_2_ FET. Red line at boundary of WSe_2_ indicates location measured with AFM, and thickness result is shown in upper right corner. (**c**) Raman spectrum by 514 nm excitation of pristine WSe_2_.

The fabrication process of the WSe_2_ FBFET is schematically shown in Figure 1a. The buried gate structure was employed for reliable gate control, and the non-uniform electrical field due to the geometrical structure of the gate was minimized. After patterning a 9 μm long trench on the 300 nm SiO_2_/Si substrate to form the buried gate, reactive-ion etching (RIE) (KVET-12000L, Korea Vacuum Tech., Daegu, Republic of Korea) was performed to form a 60 nm oxide trench pattern. To fill the gate trench, 10 nm Ti/50 nm Pt was deposited using an e-beam evaporator, and the lift-off process was performed to define the gate electrode. After the lift-off process, a chemical mechanical polishing (CMP) process was performed to level the oxide substrate and gate electrodes and remove the residual metal layer near the gate edge. Then, 30 nm thick Al_2_O_3_ was deposited as a gate dielectric using atomic layer deposition (ALD). WSe_2_ flakes (HQ graphene, Groningen, The Netherlands) were mechanically exfoliated using the Scotch tape technique and transferred to the Al_2_O_3_ layer on the targeted buried gate electrode using a dry transfer method. Plasma treatment was performed to enable p-type operation in the thick WSe_2_ flake that typically exhibits n-type characteristics. Plasma treatment was performed on the partially patterned WSe_2_ channel between the front gate and drain electrode at 20 W for 340 s in O_2_ ambient, as shown in the third step of fabrication process. The device pattern was patterned using electron beam lithography (EBL), and an electron beam evaporator was used to deposit Ti on n-type WSe_2_ and Pd on p-type WSe_2_ to form asymmetric low-resistance S/D contacts, followed by a 30 nm Al_2_O_3_ gate dielectric. Then, a highly doped 30 nm ZnO front gate layer was deposited using ALD and a 3 µm front gate pattern was formed by lithography and wet etching. For device modeling and logic circuit simulation, WSe_2_ FBFET modeling and logic circuit simulations were performed using HSPICE (Synopsys). The device characteristics of the WSe_2_ FBFET were modeled by calibrating the device parameters for a thin-film transistor model (V_th_, SS, I_on_, I_off_). Using the developed WSe_2_ FBFET model, the theoretical projections of scaled WSe_2_ FBFETs were predicted, and the gain and power consumption of scaled WSe_2_ FBFET-based logic circuits were extracted.

## 3. Results and Discussions

**Figure 2:**
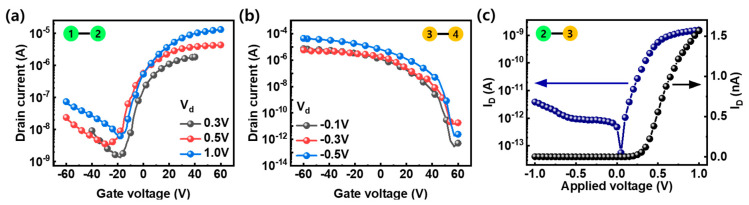
I_d_–V_g_ characteristics of FET composed of (**a**) Ti electrodes (1, 2) and the transferred pristine WSe_2_ channel; (**b**) Pd electrodes (3, 4) and O_2_ plasma-treated WSe_2_ channel. (**c**) Diode characteristics of the p–n homojunction of WSe_2_ between the Ti electrode (2) and Pd electrode (3). The numbers in the green and yellow circles on the graph represent the electrodes shown in the optical image of the device, as shown in Figure 1b.

Figure 1b shows an optical microscope image of the fabricated device. This device has electrode patterns to verify the electrical characteristics of the WSe_2_ channel. The electrodes marked 1–2, 2–3, and 3–4 shown in the optical image are used as indicators to verify the electrical characteristics of n-type WSe_2_, p–n homojunction of WSe_2_, and p-type WSe_2_, respectively. The inset in Figure 1b shows the thickness of the transferred WSe_2_ measured with an Atomic Force Microscope (AFM). The measurement location is indicated by the red line in Figure 1b. The thickness of WSe_2_ was 10.3 nm. Figure 1c shows the Raman spectrum of the WSe_2_ flake obtained by 514 nm excitation measured after the transfer. In the frequency range of 200–450 cm^−1^, three Raman peaks ($E_{2g}^{1}$, $A_{1g}$, $B_{2g}^{1}$) characterizing WSe_2_ are located at 247.8, 256.4, and 307.6 cm^−1^, respectively, and the other small peaks observed in the 350–400 cm^−1^ range are the peaks from thick WSe_2_, related to second-order and combination Raman modes [42,43]. Figure 2 shows the electrical characteristics of the device with a channel between the electrodes indicated by numbers in the optical image in Figure 1b. Figure 2a shows the I_d_–V_g_ curves of transferred pristine WSe_2_ measured at V_d_ = 0.3, 0.5, and 1.0 V. The pristine WSe_2_ FET behaved like a depletion-mode nFET with an electron current of 13 μA at V_G_ = + 60 V, V_d_ = 1.0 V. These are the typical characteristics of thick WSe_2_ above ~5 nm [44]. The plasma-treated WSe_2_ channel and Pd electrodes marked 3 and 4 show a p-type behavior where the current changes from 10^−12^ A to 10^−5^ A in the gate range from 60 V to −60 V, as shown in Figure 2b. The polarity conversion from n-type to p-type behavior after the plasma treatment process was successfully confirmed even though both devices operate in depletion mode. As shown in Figure 2c, the output curve of the device formed between the electrodes marked 2 and 3 behaves like a forward-rectifying diode and exhibits 1.6 nA at forward bias (V_d_ = 1.0 V), about 400 times higher than at reverse bias (V_d_ = −1.0 V), confirming the successful formation of a p–n homojunction in WSe_2_.

**Figure 3:**
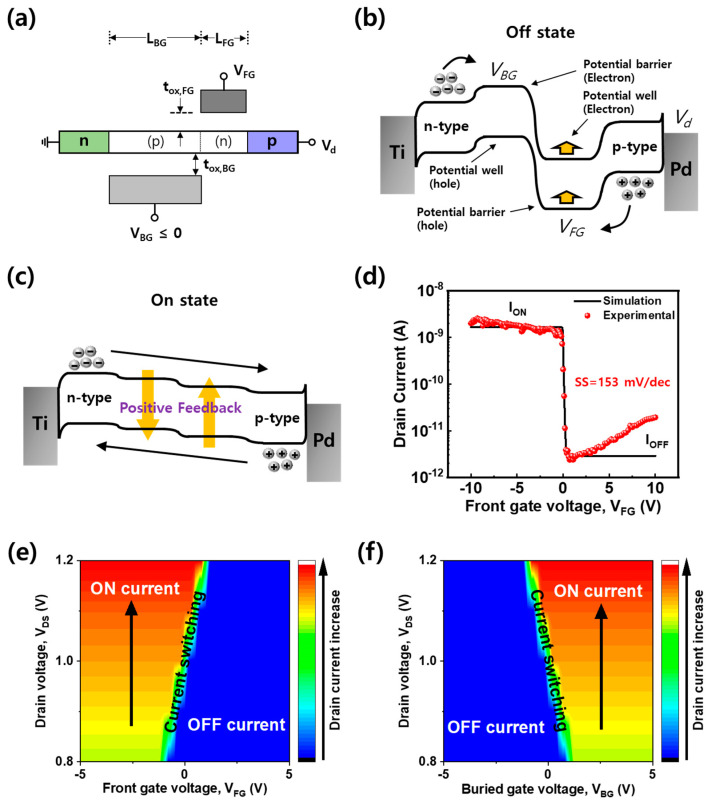
(**a**) Configuration of the WSe_2_ FBFET considering L_BG_ = 9 μm, L_FG_ = 3 μm, t_ox,FG_ = t_ox,BG_ = 30 nm. (p) and (n) are charged carrier types by band modulation. Band alignment of the (**b**) off state and (**c**) on state for the WSe_2_ FBFET. (**d**) I_d_-V_FG_ characteristics of the WSe_2_ FBFET at V_d_ = 1.0 V, V_BG_ = 0 V. The simulated correlation map of drain current between (**e**) V_FG_ and (**f**) V_BG_ at different V_d_.

A cross-sectional schematic of the WSe_2_ FBFET is shown in Figure 3a. When a forward bias is applied to the drain (Pd electrode), the forward current of the p–n junction flows from drain to source. In the off state of the FBFET, a buried gate bias (V_BG_) and a front gate bias (V_FG_) modulate the WSe_2_ channels to p-type and n-type, respectively. These gate voltages form the potential barriers and wells of holes and electrons that turn off the FBFET in the ideal band diagram, as shown in Figure 3b. When V_FG_ sweeps from the voltage forming the potential barrier to the opposite polarity (in this case, from 10 V to −10 V), the potential barrier for hole injection from drain to channel is lowered, and some of the holes are injected and accumulate in the potential well for hole. As holes are accumulated in the potential well, the potential barrier for electrons is lowered. As a result, electrons are injected into the channel from the source and accumulate in the electron potential well, further lowering the potential barrier for holes. When V_FG_ lowers the potential barrier enough to enter the feedback loop, this cycle occurs quickly and spontaneously, causing dramatic barrier modulation and switching to the on state, as shown in Figure 3c. Figure 3d shows the I_d_–V_FG_ curve measured from the WSe_2_ FBFET at V_d_ = 1.0 V, V_BG_ = 0 V. The on/off ratio is ~10^3^. The minimum SS value is 153 mV/dec. Even though this value is higher than the lower limit of silicon MOSFET ~60 mV/dec, an abrupt current increase due to the feedback mechanism at the homojunction is successfully demonstrated, even in the 2D material channel. The simulation confirmed that abrupt current switching behavior follows the feedback mechanism. Since the current switching point is affected by the barrier structure, it can vary depending on V_d_ and V_BG_. The fitting did not work for the negative-bias region because the high leakage current at the negative-bias region due to band-to-band tunneling was not included in the modeling. A simulated correlation map between the front gate bias and the drain bias is shown in Figure 3e. V_d_ determines the initial number of carriers that cannot cross the barrier formed by the front gate bias. At a high V_d_, the drain current increases, and the number of carriers crossing the barrier increases, making it easier to enter the feedback mechanism. Therefore, when V_d_ is larger than 1.0 V, current switching can occur at V_FG_ > 0 V. Figure 3f shows the simulated correlation map between the buried gate bias and the drain bias. When V_bg_ sweeps (in this case from −5 V to 5 V), the potential barrier for electrons controls the carriers, so electrons become the majority carriers. The device can be operated as an n-type feedback FET.

**Figure 4:**
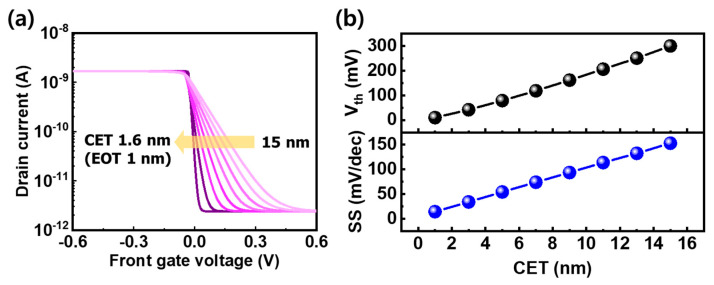
(**a**) I_d_–V_FG_ characteristics, (**b**) threshold voltage (V_th_), and subthreshold swing (SS) values for WSe_2_ FBFET when the capacitance equivalent thickness (CET) of the front gate dielectric is reduced from 15 to 1.6 nm.

**Figure 5:**
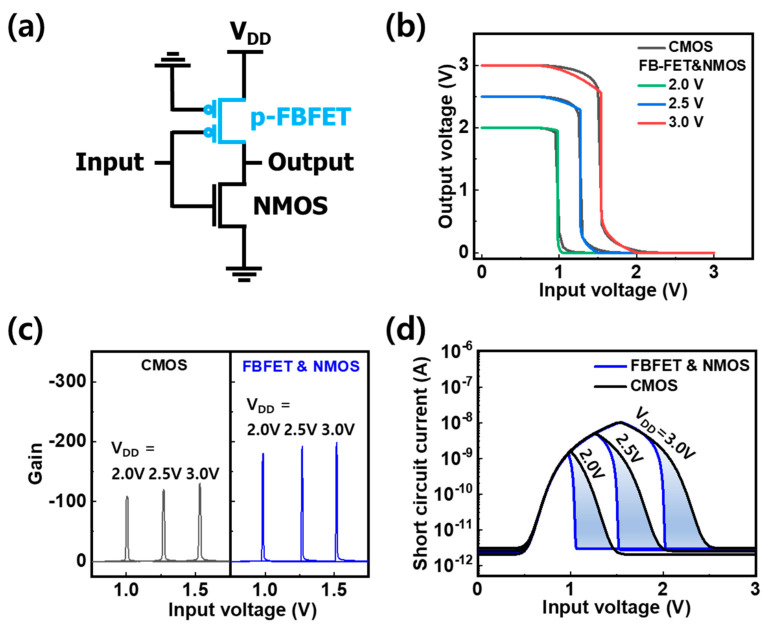
(**a**) Schematic of the inverter circuit based on WSe_2_ FBFET (p-FBFET) and n-MOSFET. Comparison of inverter characteristics composed of p-FBFET with an EOT of 1 nm and p-MOSFET with CMOS technology. (**b**) Output vs. input voltage curve. (**c**) Voltage gain vs. input voltage curve. (**d**) Short-circuit current vs. input voltage curve.

Since the high swing value of the WSe_2_ FBFET is due to the thick gate dielectric, a theoretical projection of swing was obtained using the device model fitted to the experimental data shown in Figure 3d. Figure 4a shows the simulated I–V characteristics as a function of gate dielectric thickness. The capacitance equivalent thickness (CET) of the 30 nm Al_2_O_3_ front gate dielectric is ~15 nm. As the CET of the gate dielectric targeting 1 nm of EOT is scaled down to 1.6 nm, the threshold voltage decreases to 10 mV from 300 mV, and the swing decreases to 14 mV/dec from 153 mV/dec, as shown in Figure 4b. Figure 5a shows a schematic of the inverter circuit, which consists of a pull-down WSe_2_ FBFET with a gate dielectric scaled down to 1 nm EOT (p-FBFET) and an n-type pull-up MOSFET. The n-type and p-type MOSFETs used in the simulation for the inverters had a 3 nm thick SiO_2_ gate dielectric, a length of 300 μm, and a width of 1 μm and 2 μm, respectively. Unlike the p-MOSFET, the p-FBFET, which has two gates, applies the input voltage to the front gate, which is responsible for abrupt current transition, and grounds the buried gate in the circuit configuration. In the WSe_2_ FBFET, the buried gate functions as the back gate. The inverter characteristics for supply voltages at 2.0, 2.5, and 3.0 V composed of p-FBFET were compared with an inverter using silicon CMOS technology (Figure 5b). The transfer curve of the inverter using the p-FBFET is similar to the transfer curve of a silicon CMOS inverter, but the abrupt voltage transition caused by the characteristics of the p-FBFET improves the gain of the inverter. The small-signal gain of the inverter using the FBFET was 199 at V_DD_ = 3.0 V, while that of the CMOS inverter was 130, as shown in Figure 5c. The gain of the inverter using p-FBFET was about 1.53 times higher than that of the CMOS inverter. Because of the abrupt transition, the power consumption during the state transition decreased, as shown in Figure 5d. The blue area represents the current difference between the two inverters at each supply voltage. The power consumptions of the inverter using p-FBFET and of the CMOS inverter were 5.24 nW and 5.86 nW at V_DD_ = 3.0 V, respectively, with the inverter using p-FBFET showing a difference in power consumption of −11.9% compared to the CMOS inverter. Table 1 compares the gain and power consumption between the WSe_2_ FBFET inverters at various EOTs and the CMOS inverter. When the EOT decreases from 5 nm to 1 nm, the ratio of the gain between those inverters increases to 1.53 times from 1.42 times, and the difference in power consumption is reduced to −11.9% from −3.4% compared to the CMOS inverter.

## 4. Conclusions

A FBFET using an in-plane WSe_2_ p–n homojunction has been demonstrated, and the performance of the p-FBFET has been theoretically estimated at a 1 nm EOT gate dielectric. The abrupt current switching behaviors of the WSe_2_-based p-type FBFET confirmed that the electrically modulated p–n homojunction can be used for the feedback mechanism, even in 2D materials. The theoretically projected inverter performance with a 1 nm EOT gate dielectric indicates that the gain is 1.53 times higher than that of a CMOS inverter, with a 11.9% lower power consumption. Considering that TMDC-based devices are being seriously considered for middle-of-line or backend-of-line co-integration, WSe_2_-based FBFETs are promising candidates for such applications.

## Figures and Tables

**Table 1:** Comparison between WSe_2_ FBFET and CMOS inverters for gain and power consumption at various EOTs.

Pull-Up Devices	EOT (nm)	Comparison with Performance of CMOS Inverter	
		Gain	Power Consumption
WSe_2_ FBFET	1	1.53 times	−11.9%
	3	1.44 times	−6.8%
	5	1.42 times	−3.4%

This table is a performance comparison at V_DD_ = 3.0 V.

## Data Availability

Data are contained within the article.
